# Use of iStent as a Standalone Operation in Patients with Open-Angle Glaucoma

**DOI:** 10.1155/2020/8754730

**Published:** 2020-05-24

**Authors:** Yu-Yen Chen, Yun-Ju Lai, Yung-Feng Yen, Li-Ying Huang

**Affiliations:** ^1^School of Medicine, National Yang-Ming University, Taipei 112, Taiwan; ^2^Department of Ophthalmology, Taichung Veterans General Hospital, Taichung 407, Taiwan; ^3^School of Medicine, Chung Shan Medical University, Taichung 402, Taiwan; ^4^Community Medicine Research Center and Institute of Public Health, National Yang-Ming University, Taipei 112, Taiwan; ^5^Division of Endocrinology and Metabolism, Department of Internal Medicine, Puli Branch of Taichung Veterans General Hospital, Nantou 545, Taiwan; ^6^Department of Exercise Health Science, National Taiwan University of Sport, Taichung 404, Taiwan; ^7^Section of Infectious Diseases, Taipei City Hospital, Taipei 103, Taiwan; ^8^Institute of Hospital and Health Care Administration, National Yang-Ming University, Taipei 112, Taiwan; ^9^Department of Health Care Management, National Taipei University of Nursing and Health Sciences, Taipei 112, Taiwan; ^10^School of Medicine, College of Medicine, Fu Jen Catholic University, New Taipei 242, Taiwan; ^11^Division of Endocrinology and Metabolism, Department of Internal Medicine, Fu Jen Catholic University Hospital, New Taipei 243, Taiwan; ^12^Department of Medical Education, Fu Jen Catholic University Hospital, New Taipei 243, Taiwan

## Abstract

**Purpose:**

The iStent provides a direct pathway for aqueous outflow from the anterior chamber to Schlemm's canal in patients with open-angle glaucoma (OAG). We performed a meta-analysis to evaluate the effectiveness of iStent as a standalone operation in patients with OAG in reducing the intraocular pressure (IOP) and the number of topical glaucoma medications.

**Methods:**

We searched various databases between January 1, 2000, and September 30, 2019, and included only peer-reviewed, prospective, or retrospective clinical studies in our analyses. Details regarding the IOP and the number of medications at baseline and end point were recorded from each study. Standardized mean differences (SMDs) of IOP and medication numbers were calculated. Furthermore, the success rate (the proportion of IOP ≤18 mmHg and IOP reduction ≥20% at end point) and the complication rate were also summarized. Finally, a subgroup analysis was done based on the iStent generation (first and second), follow-up duration (≤6, 6–18, 18–36, and >36 months), and iStent number (one, two, and three). The outcome measures were aggregated SMDs computed from each study.

**Results:**

A total of 17 studies with 978 eyes were included in this analysis. All studies demonstrated a reduction in IOP after iStent implantation. Aggregated SMDs of IOP revealed a significant reduction (SMD = −2.64, 95% confidence interval (CI): −3.21 to −2.07). The success rate was significantly good, and most of the complication rates were low. The number of medications was also significantly reduced (SMD = −1.71, 95% CI: −2.18 to −1.24). The subgroup analysis revealed a reduction in IOP and medication burden in each category of iStent generation, follow-up duration of up to 42 months, and iStent numbers.

**Conclusion:**

Use of iStent as a standalone procedure does reduce the IOP and the number of glaucoma medications. The benefit of iStent lasts for at least 42 months.

## 1. Introduction

Glaucoma is a leading cause of irreversible blindness. Open-angle glaucoma (OAG) is the most prevalent form of glaucoma, and the first-line management consists of medications that reduce the intraocular pressure (IOP). When a medication leads to severe side effects or does not reduce the IOP to a satisfied extent, surgical intervention is indicated. Trabeculectomy has been the mainstay of filtering surgery for decades; however, scar formation after the operation may impede the aqueous outflow. Microinvasive glaucoma surgery (MIGS), which has the advantages of minimal manipulation and less postoperative fibrous tissue proliferation, has emerged recently. The iStent is a trabecular microbypass stent (Glaukos Corporation, San Clemente, California) and is one of the first MIGS devices applied in patients with OAG.

Previous studies have investigated the safety and efficacy of iStent and demonstrated promising results [1–10]. However, the majority of studies had small case numbers and interstudy variations in the follow-up time and outcome assessment. A practical method to solve these problems is to perform a meta-analysis to derive pooled results. However, the majority of meta-analyses investigating iStent have evaluated its use as an adjunctive therapy in cataract surgery for patients with both cataract and OAG [11–14], and only a very few studies have investigated its effect when used as a standalone procedure [15, 16]. In an earlier study, Malvankar-Mehta et al. reported a 22% reduction in IOP from baseline at 18 months after one iStent implantation. They also observed a significant decrease in the number of topical glaucoma medications after iStent implantation [15]. Some clinical studies investigating iStent implantation as a standalone operation were published later on and must be included in statistical analyses.

Therefore, we conducted this meta-analysis incorporating additional studies. Our aim was to analyze the available data on the use of iStent as a standalone procedure to summarize its effect on patients with OAG.

## 2. Materials and Methods

### 2.1. Search Strategy

This study was conducted according to the Preferred Reporting Items for Systematic Reviews and Meta-Analyses (PRISMA) guidelines. We searched the PubMed, EMBASE, and Cochrane databases for studies published from January 1, 2000, to September 30, 2019, using the keywords “glaucoma” and “iStent.” Studies were screened first by examining the titles and abstracts and then by scrutinizing full texts. Bibliographies were also manually searched for the relevant literature.

### 2.2. Inclusion and Exclusion Criteria

Only peer-reviewed journal articles written in English were included in this study. They should be original, prospective, or retrospective clinical studies investigating the effect of standalone iStent implantation for at least five eyes. Reviews, meta-analyses, or conference abstracts were excluded because of repeated data. Two researchers (Chen and Lai) independently assessed the articles. A third researcher (Yen) intervened if consensus was not reached.

### 2.3. Data Extraction

The following data were recorded from each included article: the first author, year of publication, age of participants, iStent generations, number of iStents implanted, follow-up duration, sample size, baseline IOP, end point IOP (IOP at the final follow-up), and the number of topical glaucoma medications at baseline and end point.

### 2.4. Statistical Analysis

The meta-analysis was performed using the Comprehensive Meta-Analysis software, version 3 (Biostat, Eaglewood, NJ, USA). One of the outcomes was the standardized mean difference (SMD) of IOP between the end point and baseline. To compute the SMD from each study, the mean difference between the end point and baseline was divided by the standard deviation to ensure that the difference was on the same scale in each study. Another outcome was the SMD of the number of medications. Using a similar algorithm, the SMD of the number of glaucoma medications between the end point and baseline was derived. The SMDs from each study were then pooled to derive the overall value.

Subsequently, between-trial heterogeneity was determined using *I*^2^ statistics. An *I*^2^ statistics of ≥50% represents high heterogeneity. Funnel plots and Egger's test were used to assess publication bias.

Thereafter, efficacy (success rate) and safety of iStent implantation were evaluated. Proportion of patients with a mean IOP of 18 mmHg or less and proportion of patients who experienced a mean reduction in IOP of 20% or more at the end point stand for the success rate of iStent implantation. We also recorded the complication events and rates in each study to assess the safety of iStent implantation.

Finally, a subgroup analysis was performed to examine the impact of iStent generations (first generation vs. second generation), follow-up duration (≤6, 6–18, 18–36, and >36 months), and iStent numbers (one, two, and three) on the outcomes.

## 3. Results

### 3.1. Search Results and Characteristics of Included Studies

**Figure 1:**
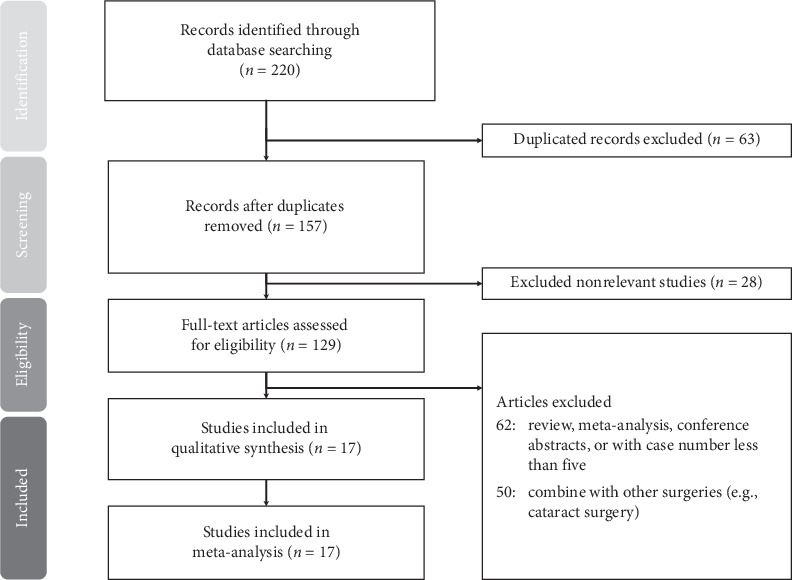
Preferred Reporting Items for Systematic Reviews and Meta-Analyses (PRISMA) flow diagram for the searching and identification of included studies.

Figure 1 shows the PRISMA flow diagram. First, we retrieved 220 citations. After eliminating duplicated records (*n* = 63), there were 157 studies. Then, we excluded nonrelevant studies (*n* = 28). Those categorized as reviews, meta-analyses, conference abstracts, or with case number less than five were also excluded (*n* = 62). Studies investigating iStent but combined with other surgeries (e.g., cataract surgery) or with prior incisional surgeries were also removed from the lists (*n* = 50). Finally, 17 studies were enrolled in our meta-analysis.

Table 1 summarizes the characteristics of the included studies. A total of 978 eyes were evaluated in the 17 studies. In the majority of studies, the mean age of the participants was 60–70 years. First-generation iStent was used in 599 (61.2%) eyes, and second-generation iStent was used in 379 (38.8%) eyes. The follow-up duration was 6 months in 3 studies, 12 months in 3 studies, 18 months in 5 studies, 24 months in 1 study, 36 months in 4 studies, and 42 months in 1 study. Regarding the number of iStents, 159 (16.3%) eyes had 1 stent, 739 (75.5%) had 2 stents, and 80 (8.2%) eyes had 3 stents.

### 3.2. Outcome Assessment

**Figure 2:**
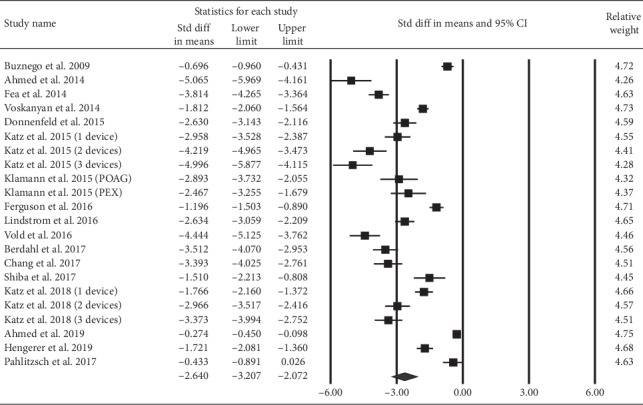
The overall effect of iStent on intraocular pressure.

Figure 2 illustrates the SMDs of IOP between the end point and baseline. All studies reported a reduction in IOP after iStent implantation. Pooled result demonstrated a significant SMD of −2.64 (95% confidence interval (CI): −3.21 to −2.07).

**Figure 3:**
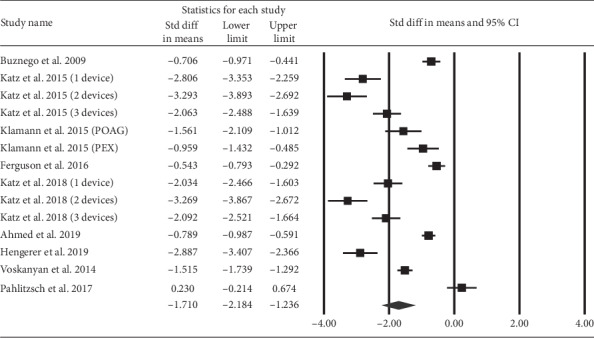
The overall effect of iStent on the number of glaucoma medications.

The SMDs of the number of topical glaucoma medications are illustrated in Figure 3. The majority of studies reported a reduction in the number of medications at the end point compared to that at baseline. The overall SMD was −1.71 (95% CI: −2.18 to −1.24).

### 3.3. Heterogeneity and Publication Bias

**Figure 4:**
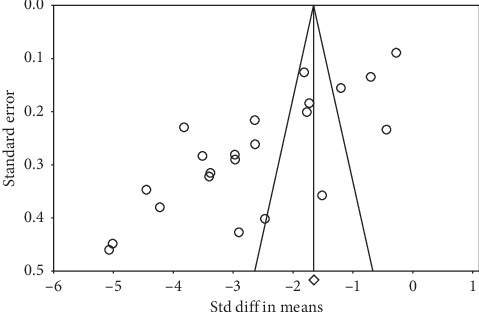
Funnel plot of studies regarding iStent on IOP. IOP: intraocular pressure.

**Figure 5:**
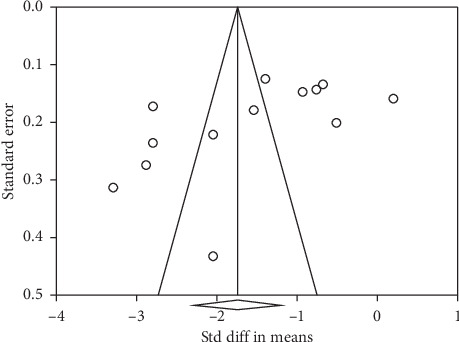
Funnel plot of studies regarding iStent on the number of medications.

There was a moderate heterogeneity in studies evaluating the SMDs of IOP as well as the number of medications (*I*^2^ = 97.4% and 95.9%, respectively). Figures 4 and 5 present the results of Egger's test (*p* < 0.001 and *p*=0.01, respectively).

### 3.4. Success Rate and Complication Rate of iStent Implantation

**Figure 6:**
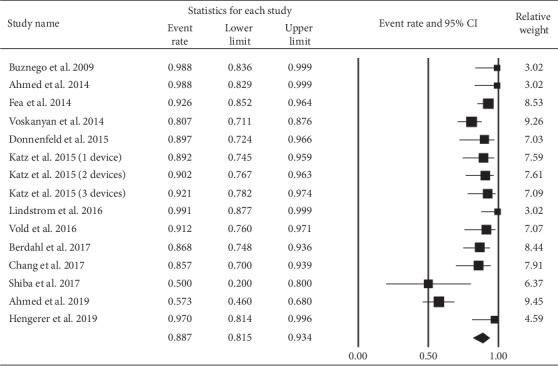
Proportion of eyes with an IOP ≤18 mmHg at the end point.

**Figure 7:**
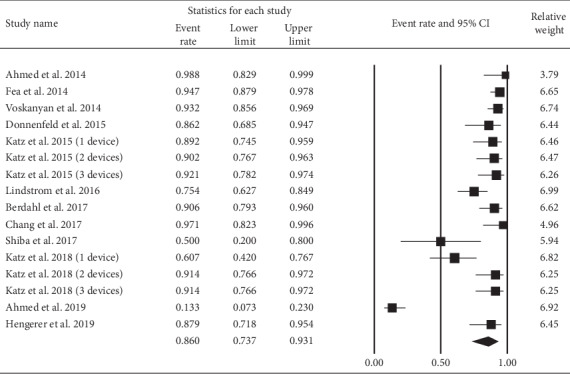
Proportion of eyes with an IOP reduction ≥20% at the end point.

In Table 2, we assessed the proportions of IOP ≤18 mmHg and IOP reduction ≥20% at the end point. Both of them revealed significant effects.  Figure 6 shows that IOP ≤18 mmHg at the end point was achieved by 88.7% (95% CI: 81.5% to 93.4%) of eyes.  Figure 7 illustrates 86.0% (95% CI: 73.7% to 93.1%) of eyes had a 20% or greater reduction in IOP at the end point.

The complication events and rates of iStent implantation are summarized in Table 2. Most of the studies had a complication rate of less than 20%; however, some studies had more occurrences of complications. The variation resulted from different definitions regarding complications. Minor, transient complications were included in the calculation of complication rates in some, but not in all studies. Since the complication events reported in different studies were not based on the same criteria, they could not be compared directly. However, we still could find out that the more frequent complications were progression of cataract and elevated IOP. iStent obstruction or iStent malposition (not visible) were not common and only occurred in two and three studies, respectively.

### 3.5. Subgroup Analyses

**Figure 8:**
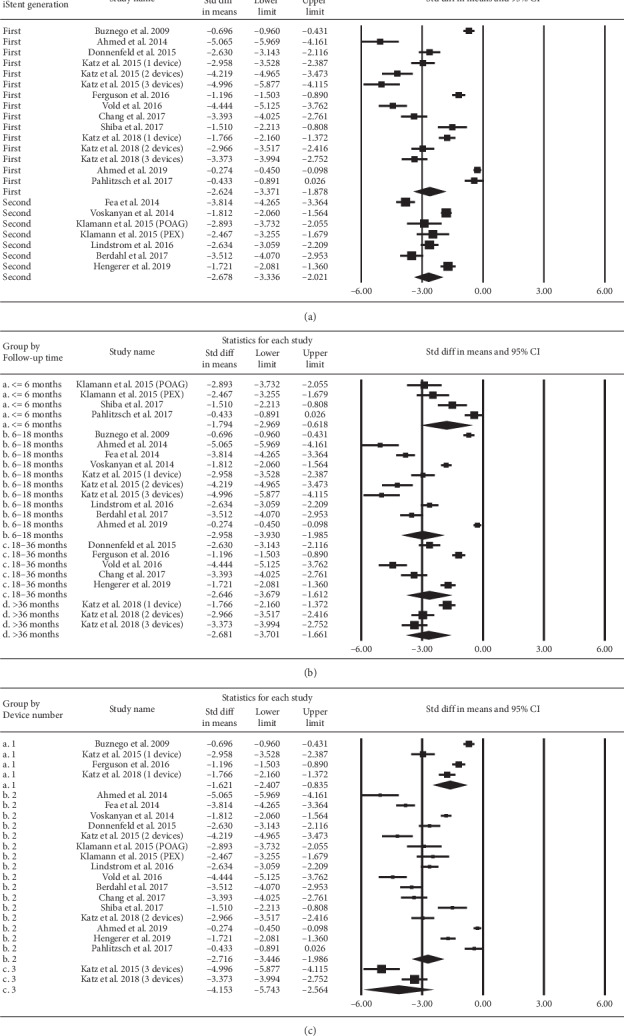
Subgroup analysis regarding the effect of iStent on intraocular pressure, stratified by (a) iStent generation, (b) follow-up time, and (c) iStent numbers.

**Figure 9:**
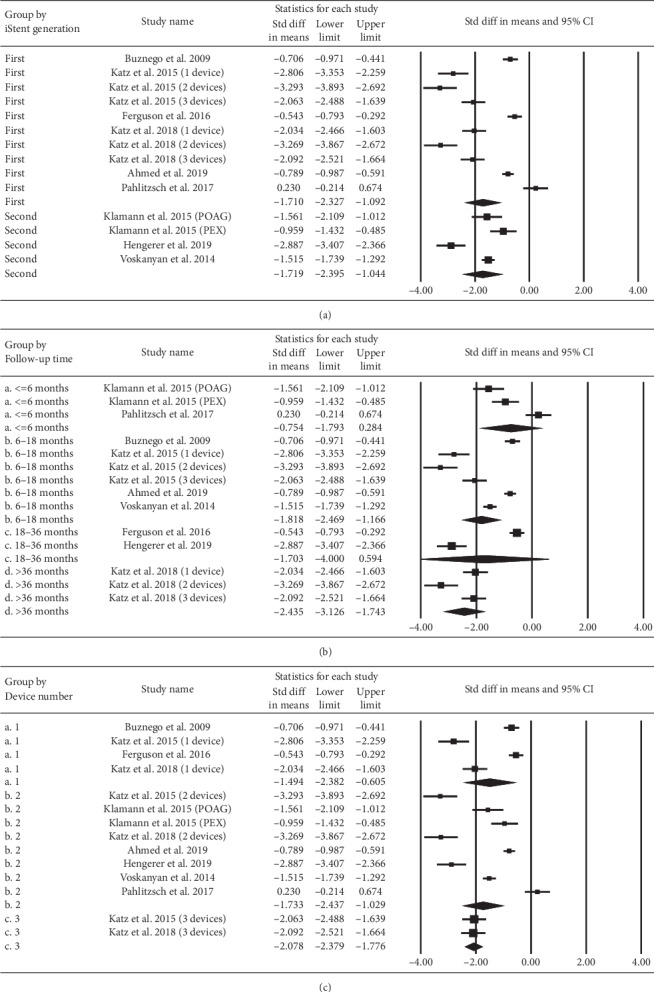
Subgroup analysis regarding the effect of iStent on the number of medications, stratified by (a) iStent generation, (b) follow-up time, and (c) iStent numbers.

The SMDs of IOP stratified according to iStent generation, follow-up duration, and iStent numbers are depicted in Figure 8. Both the first- and the second-generation iStents demonstrated a significant reduction in IOP. A similar result was observed for the subgroups of different follow-up duration (up to 42 months) and different iStent numbers. A higher number of iStents could reduce more IOP.  Figure 9 displays the results of similar subgroup analyses regarding the pooled SMDs of the number of medications. Both the first- and the second-generation iStents significantly reduced the number of medications. Eyes in all categories of follow-up duration showed a reduction in the point estimate for medication use. The extent of reduction was significant at the follow-up duration of 6–18 and 42 months. Regarding the number of iStents, one or two or three iStents significantly reduced the number of medications.

## 4. Discussion

This meta-analysis focused on the impact of using iStent in reducing the IOP and the number of glaucoma medications using 17 studies derived from the database search. Between the end point and the baseline, the SMD of IOP was −2.64 (95% CI: −3.21 to −2.07) and that of the number of medications was 1.71 (95% CI: −2.18 to −1.24). Subgroup analyses also revealed a reduction in IOP and the number of medications when stratified according to iStent generation, follow-up duration, and iStent numbers.

The iStent reduces IOP by providing a direct pathway for aqueous outflow from the anterior chamber to Schlemm's canal. The first-generation iStent was approved by the Food and Drug Administration (FDA) in 2012, and the second-generation iStent received FDA's approval in 2018. Both these iStents are indicated for use in conjunction with cataract surgery for patients with OAG. There are limited data regarding the effect of iStent as a standalone procedure. The strength of our study is that we analyzed the outcome of standalone iStent implantation, and hence we were able to derive the “pure” effect of iStent on the IOP and the number of medications. We found a significant reduction in the IOP and the number of medications after the standalone procedure of iStent implantation. These findings were similar to those of previous meta-analyses conducted by Malvankar-Mehta and Patel [15, 16]. Furthermore, in our meta-analysis, most of the complications had a rate of less than 20%. Only two studies had iStent obstruction, and three studies had iStent malposition. Therefore, iStent implantation is not only effective but also safe if performed properly.

Another strength of our study is the subgroup analyses are performed according to iStent generation, follow-up duration, and number of iStents. Our findings are consistent with those of the study conducted by Malvankar-Mehta in terms of the effectiveness of iStent generation (first and second), follow-up duration (≤6, 6–18, 18–36, and >36 months), and iStent numbers (one, two, and three) [15]. The studies with the longest follow-up duration included in our meta-analysis reported that the benefit of iStent persisted for up to 42 months. Such a sustained long-term IOP-reducing effect suggests that iStent can be used as a suitable treatment option for patients with OAG. Regarding the number of iStents, we found that more iStents would reduce more IOP. However, one stent alone could also still significantly reduce the IOP.

A limitation of our analyses is the substantial heterogeneity among the included studies. The heterogeneity was multifactorial and may have been caused due to discrepancies in the study population, demographics, study location, surgeon's experience, severity of glaucoma, and baseline IOP/medication numbers. Despite the presence of heterogeneity among the studies, almost all of them demonstrated a tendency toward lower IOP/medication numbers, thereby providing evidence for a promising effect of iStent. However, we should still meticulously interpret the results because of the publication bias. Since iStent is currently in an early stage, clinical trials are largely sponsored by industries, possibly leading to publication bias. Further research from hospitals or academic institutes is required to advance our understanding of the effect of iStent implantation.

Another limitation is that all the enrolled patients were diagnosed with OAG, which consists of not only “pure” primary open-angle glaucoma (POAG), pseudoexfoliation glaucoma, and pigmentary glaucoma. Almost every study reported the overall outcome in these patients as a whole, not specifically in any subtypes of OAG. Thus, we could not know the efficacy and safety of iStent implantation in “pure” POAG eyes. Further clinical studies may be needed to focus on the pure glaucoma forms.

In conclusion, this meta-analysis has demonstrated that using iStent as a standalone procedure can reduce the IOP and the number of medications in patients with OAG. It also highlights the benefits of using iStent for patients with OAG in terms of each iStent generation, follow-up duration of up to 42 months, and iStent numbers of one to three. These findings suggest that using iStent as a standalone procedure can be potentially highly relevant in terms of clinical aspects and public health perspective. As the number of patients with OAG requiring the iStent procedure increases, further studies would become available to draw more precise conclusions in the future.

## Figures and Tables

**Table 1:** Characteristics of patients in studies included in meta-analysis.

Author, year	Age (years)	iStent generation	iStent number	Follow-up (months)	Number of eyes	Baseline IOP (mmHg)	End point IOP (mmHg)	Number of baseline medications	Number of end point medications	Reduction in IOP (mmHg)	Reduction in number of medications
Buznego, 2008 [17]	NR	First	1	18	41	21.5 ± 6.9	16.7 ± 6.9	1.6 ± 1.7	0.4 ± 1.7	4.8	1.2
Ahmed et al., 2014 [18]	62.8 ± 12.6	First	2	18	39	22.2 ± 2.0	11.8 ± 2.1	2 ± 0	1 ± 0	10.4	1 ± 0
Fea et al., 2014 [19]	64.5 ± 10.3	Second	2	12	94	21.1 ± 1.7	13.0 ± 2.3	1 ± 0	0.09 ± 0.40	8.1 ± 2.6	0.91 ± 0.40
Voskanyan et al., 2014 [20]	66.4 ± 10.9	Second	2	12	99	22.1 ± 3.3	15.7 ± 3.7	2.21 ± 0.44	NR	6.4	1.78 ± 0.91
Donnenfeld et al., 2015 [21]	66.7 ± 10.0	First	2	36	39	20.6 ± 2.0	15.2 ± 2.1	1 ± 0	NR	5.5 ± 2.7	NR
Katz et al., 2015 [22]	68.1 ± 9.1	First	1	18	38	19.8 ± 1.3	15.6 ± 1.5	1.71 ± 0.61	0.111 ± 0.5	4.2	1.6
	67.8 ± 9.3	First	2	18	41	20.1 ± 1.6	13.8 ± 1.3	1.76 ± 0.54	0.121 ± 0.394	6.3	1.64
	60.9 ± 8.1	First	3	18	40	20.4 ± 1.8	12.1 ± 1.2	1.51 ± 0.69	0.079 ± 0.269	8.3	1.50
Klamann et al., 2015 [23]	61.67 ± 5.15	Second	2	6	17 (POAG)	21.19 ± 2.56	14.19 ± 1.38	2.19 ± 0.91	0.88 ± 0.62	7.0	1.31
	62.76 ± 4.81	Second	2	6	15 (PEX)	23.75 ± 3.28	15.33 ± 1.07	2.33 ± 1.23	1.04 ± 0.30	8.42	1.29
Ferguson et al., 2016 [24]	80.07 ± 8.54	First	1	24	42	20.26 ± 6.00	13.62 ± 4.55	1.95 ± 1.01	1.33 ± 1.22	6.64	0.62
Lindstrom et al., 2016 [25]	65.3 ± 9.0	Second	2	18	57	19.5 ± 1.5	14.4 ± 2.1	1 ± 0	0.02 ± 0.13	5.1	0.98
Vold et al., 2016 [26]	64.5 ± 11.1	First	2	36	54	25.5 ± 2.5	14.6 ± 2.4	0 ± 0	0.21 ± 0.54	10.9	0.79
Berdahl et al., 2017 [27]	64.7 ± 9.6	Second	2	18	53	19.7 ± 1.5	12.9 ± 2.1	2 ± 0	1 ± 0	6.8	1 ± 0
Chang et al., 2017 [28]	62.8 ± 12.6	First	2	36	39	22.4 ± 2.3	14.0 ± 2.6	2 ± 0	1 ± 0	8.4	1 ± 0
Pahlitzsch et al., 2017 [29]	68.6 ± 16.4	First	2	6	20	21.3	16.0	2.45	2.5	5.3	Increase 0.05
Shiba et al., 2017 [30]	64.6 ± 10.7	First	2	6	10	22.0 ± 3.0	16.9 ± 3.6	NR	NR	5.1	NR
Katz et al., 2018 [31]	68.1 ± 9.1	First	1	42	38	19.8 ± 1.3	15.0 ± 2.8	1.71 ± 0.61	0.55 ± 0.50	4.8	1.16
	68.1 ± 9.1	First	2	42	41	20.1 ± 1.6	15.7 ± 1.0	1.76 ± 0.54	0.105 ± 0.307	4.4	1.66
	68.1 ± 9.1	First	3	42	40	20.4 ± 1.8	14.8 ± 1.3	1.53 ± 0.69	0.08 ± 0.27	5.6	1.45
Ahmed et al., 2019 [32]	66.5 ± 9.5	First	2	12	77	19.1 ± 3.6	18.1 ± 3.7	2.7 ± 0.8	1.69 ± 1.37	1.0	1.01
Hengerer et al., 2019 [33]	71.3 ± 10.5	Second	2	36	44	25.3 ± 6.0	14.6 ± 2.0	2.98 ± 0.88	0.55 ± 0.79	10.7	2.43

PEX = pseudoexfoliation glaucoma.

**Table 2:** Success rate and complications of studies included in meta-analysis.

Author, year	Number of eyes at baseline	Number of eyes at end point	Success rate		Complication rate	
			IOP ≤18 mmHg (*n*, %)	IOP reduction ≥20% (*n*, %)	Event (*n*, %)	Types of complication (%)
Buznego, 2008 [17]	41	41	41 (100)	NR	6 (15)	Malposition (15)
Ahmed et al., 2014 [18]	39	39	39 (100)	39 (100)	0 (0)	None
Fea et al., 2014 [19]	94	94	87 (92.6)	89 (94.7)	3 (3)	IOP decompensation (1), one stent not visible (1), soreness/discomfort (1)
Voskanyan et al., 2014 [20]	99	88	71 (80.7)	82 (93.2)	35 (35.3)	Elevated IOP (10.1), iStent obstruction (3), progression of cataract (1), allergic reaction to medications (1), iStent malposition (1), intraocular inflammation (1), subconjunctival hemorrhage (1), iStent not visible upon gonioscopy (13.1), posterior capsular opacification (2), goniosynechiae (1), lens-iris synechiae (1)
Donnenfeld et al., 2015 [21]	39	29	26 (89.7)	25 (86.2)	10 (25.6)	Progression of cataract (7.7), death (5.1), hyphema (2.6), initial cataract (2.6), proliferative diabetic retinopathy (2.6), scar from age-related macular degeneration (2.6), cataract surgery (2.6)
Katz et al., 2015 [22]	38 (1 device)	37	33 (89.2)	33 (89.2)	2 (5.3)	Cataract surgery (5.3)
	41 (2 devices)	41	37 (90.2)	37 (90.2)	0 (0)	None
	40 (3 devices)	38	35 (92.1)	35 (92.1)	2 (5.3)	Cataract surgery (5.3)
Klamann et al., 2015 [23]	17 (POAG)	17	NR	NR	32 (91.4)	Intraoperative blood reflux (91.4)
	15 (PEX)	15	NR	NR		
	3 (PG)	3	NR	NR		
Ferguson et al., 2016 [24]	42	42	NR	NR	1 (2.4)	Elevated IOP (2.4)
Lindstrom et al., 2016 [25]	57	57	57 (100)	43 (75.4)	1 (1.8)	Progression of cataract (1.8)
Vold et al., 2016 [26]	54	34	31 (91.1)	NR	12 (22.2)	Progression of cataract (20.4), hyphema (1.8)
Berdahl et al., 2017 [27]	53	53	46 (86.8)	48 (90.6)	0 (0)	None
Chang et al., 2017 [28]	39	35	30 (85.7)	34 (97.1)	13 (33.3)	Transient hypotony (5.1), progression of cataract (23.1), insomnia/malaise (2.6), herpetic corneal ulcer (2.6)
Pahlitzsch et al., 2017 [29]	20	20	NR	NR	NR	NR
Shiba et al., 2017 [30]	10	8	4 (50)	4 (50)	9 (90)	Hyphema (40), elevated IOP (10), peripheral anterior synechiae (40)
Katz et al., 2018 [31]	38 (1 device)	28	NR	17 (60.7)	8 (21.1)	Progression of cataract (21.1)
	41 (2 devices)	35	NR	32 (91.4)	5 (12.2)	Progression of cataract (12.2)
	40 (3 devices)	35	NR	32 (91.4)	7 (17.5)	Progression of cataract (17.5)
Ahmed et al., 2019 [32]	77	75	43 (57.3)	10 (13.3)	15 (19.5)	Elevated IOP (5.2), new cataract (1.3), iStent obstruction (13.0)
Hengerer et al., 2019 [33]	44	33	32 (97.0)	29 (87.9)	4 (9.1)	Mild hyphema (2.3), progression of cataract (4.5), uveitis (2.3)

## Abbreviations

OAG: Open-angle glaucoma
IOP: Intraocular pressure
MIGS: Microinvasive glaucoma surgery
PRISMA: Preferred Reporting Items for Systematic Reviews and Meta-Analyses
SMD: Standardized mean difference
CI: Confidence interval
POAG: Primary open-angle glaucoma
PEX: Pseudoexfoliation glaucoma
PG: Pigmentary glaucoma
FDA: Food and Drug Administration.
